# Supplementary material for: Deep mutational learning for the selection of therapeutic antibodies resistant to the evolution of Omicron variants of SARS-CoV-2
Source: Nat Biomed Eng. 2025 Mar 5;9(4):552–65. doi: 10.1038/s41551-025-01353-4 (PMC12003156; doi:10.1038/s41551-025-01353-4)
Supplement: Supplementary file 1 — Supplementary Figs. 1–10 and Tables 1–6. [file 41551_2025_1353_MOESM1_ESM.pdf]

nature biomedical engineering

Supplementary information

https://doi.org/10.1038/s41551-025-01353-4

Deep mutational learning for the selection of therapeutic antibodies resistant to the evolution of Omicron variants of SARS-CoV-2

In the format provided by the authors and unedited

Supplementary figures

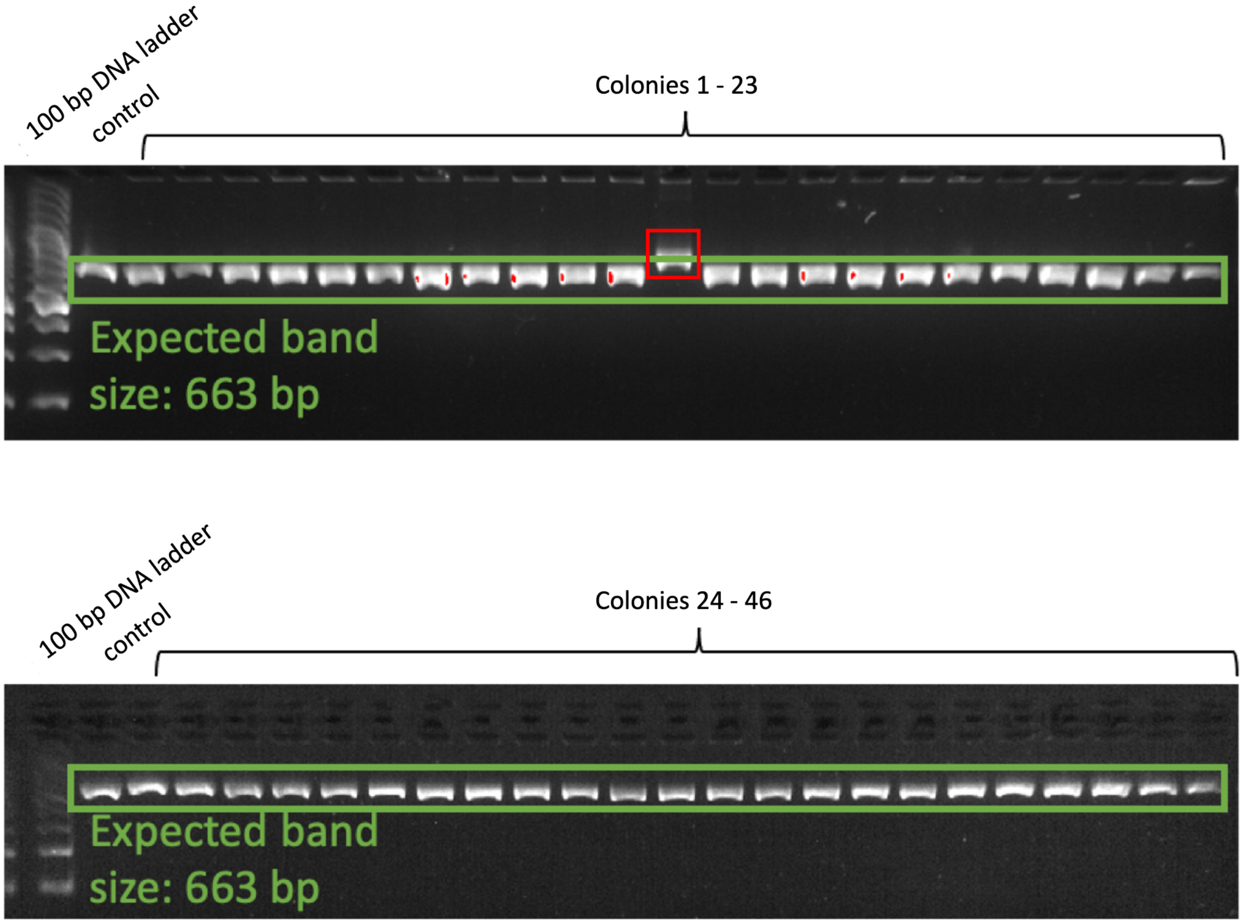

Supplementary Fig. 1 I After assembling the RBD sequence from short fragments and transformation into *E. coli*, single colonies were picked and colony PCRs (cPCR) were performed. For the amplification, primers binding directly upstream and downstream of the RBD were used. As a control, WT BA.1 plasmid was used. When running the cPCR products on a 2% agarose gel, 45 out of 46 reactions showed the right band size of 663 base pairs (bp) (wrongly assembled variant highlighted in red), roughly corresponding to 98% correctly assembled full length RBD sequences.

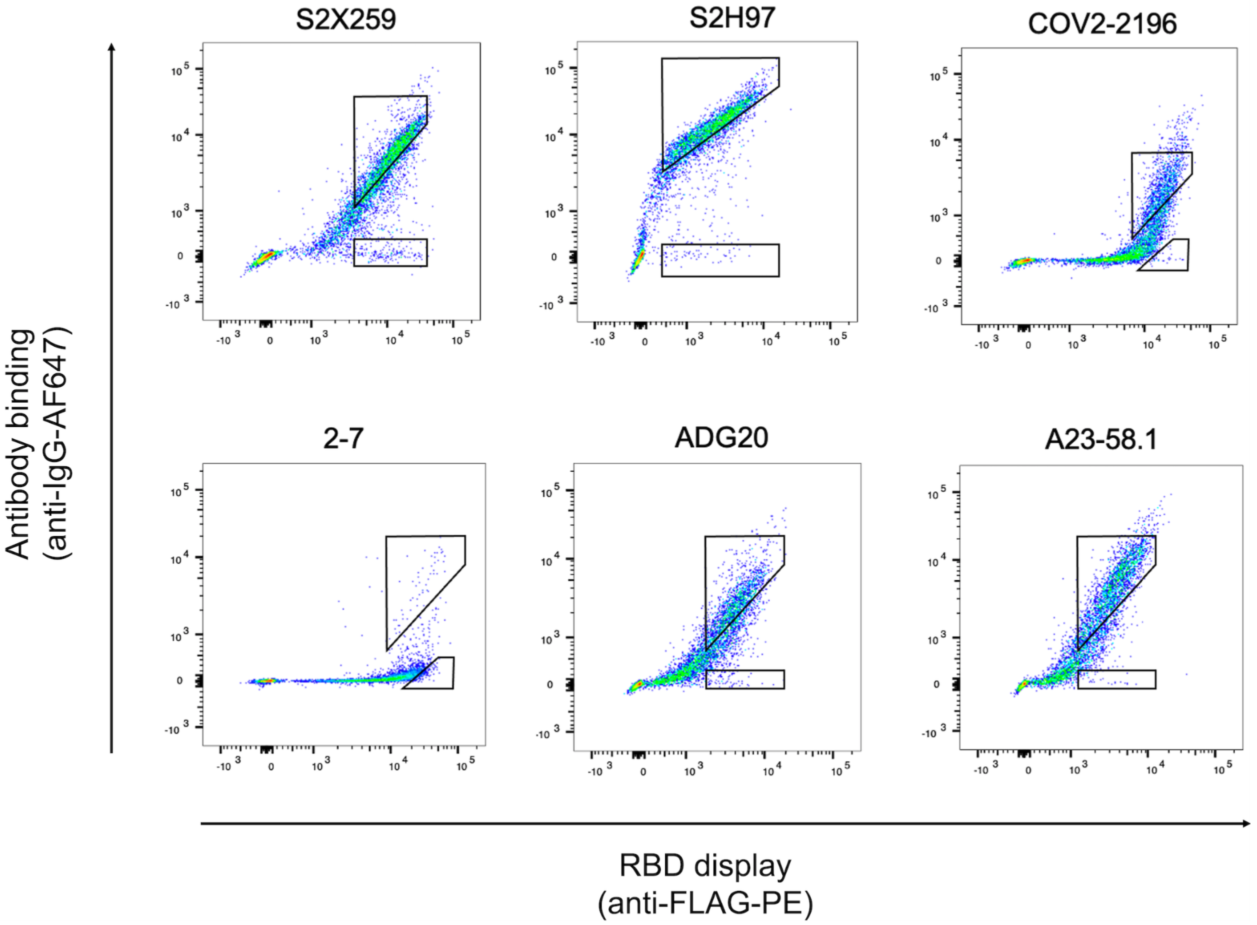

Supplementary Fig. 2 l Representative FACS dot plots of yeast RBD libraries during antibody screening; sorting gates for binding and non-binding (escape) populations are shown.

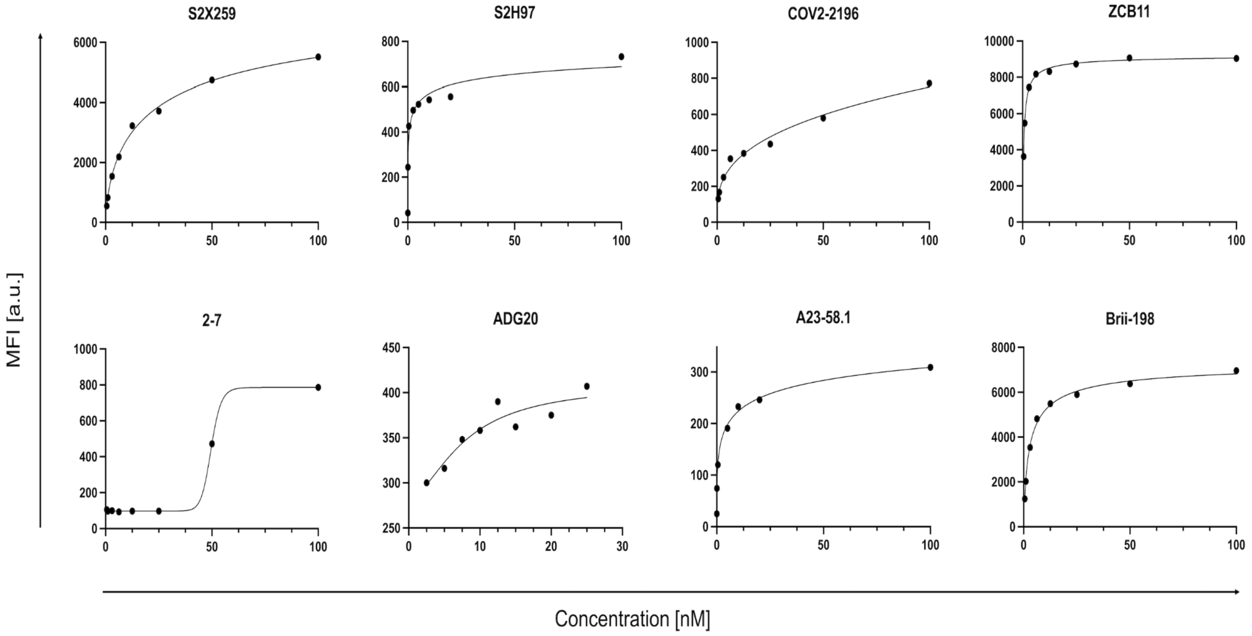

Supplementary Fig. 3 | Titration curves of individual antibodies tested against yeast-displayed Omicron BA.1 RBD. The mean fluorescent intensity (MFI) is shown as a function of antibody concentration.

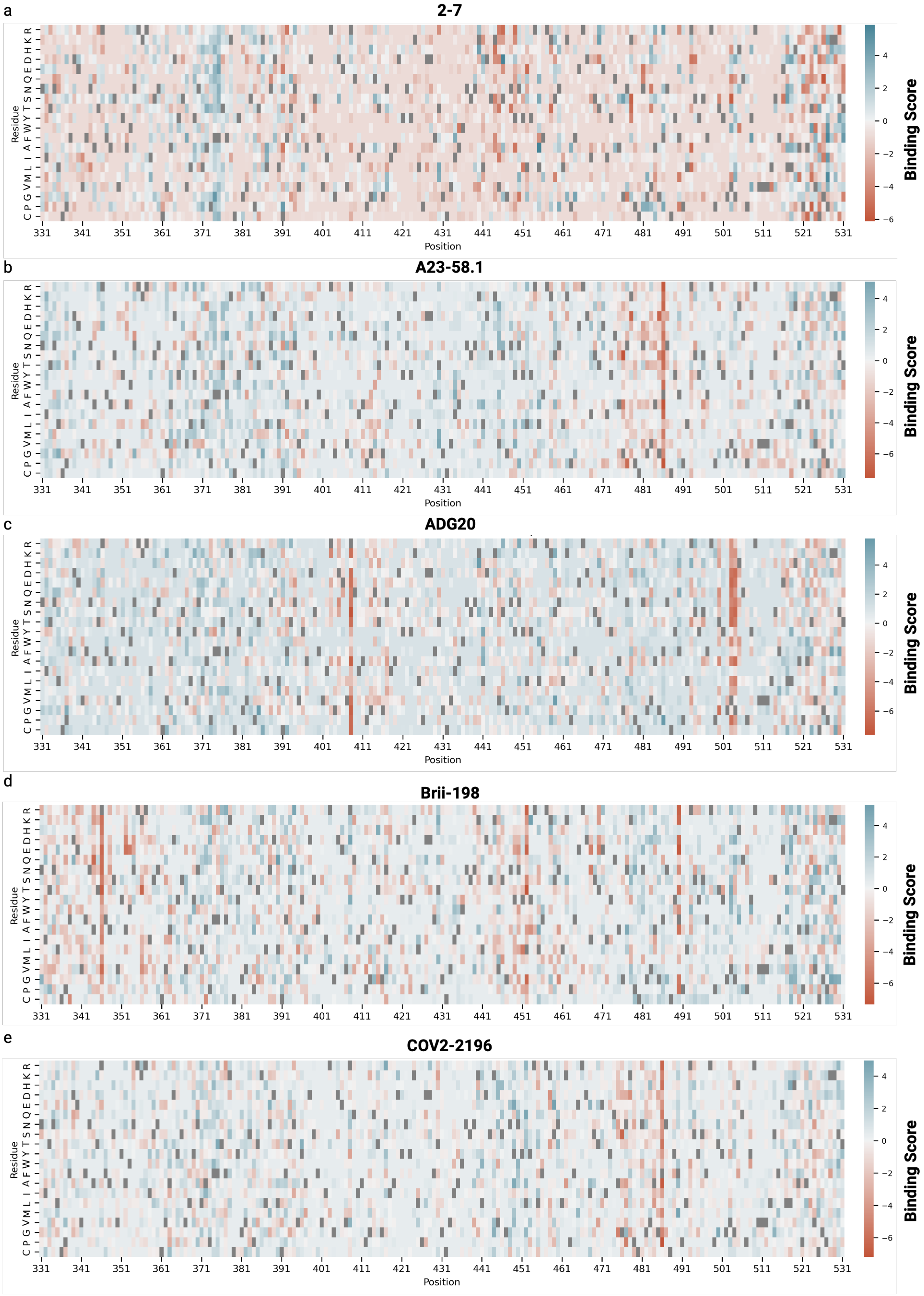
a
2-7
b
A23-58.1
c
ADG20
d
Brii-198
e
COV2-2196
Residue
C P G V M L I A F W Y T S N Q E D H K R
Position
331 341 351 361 371 381 391 401 411 421 431 441 451 461 471 481 491 501 511 521 531
Binding Score
4 2 0 −2 −4 −6

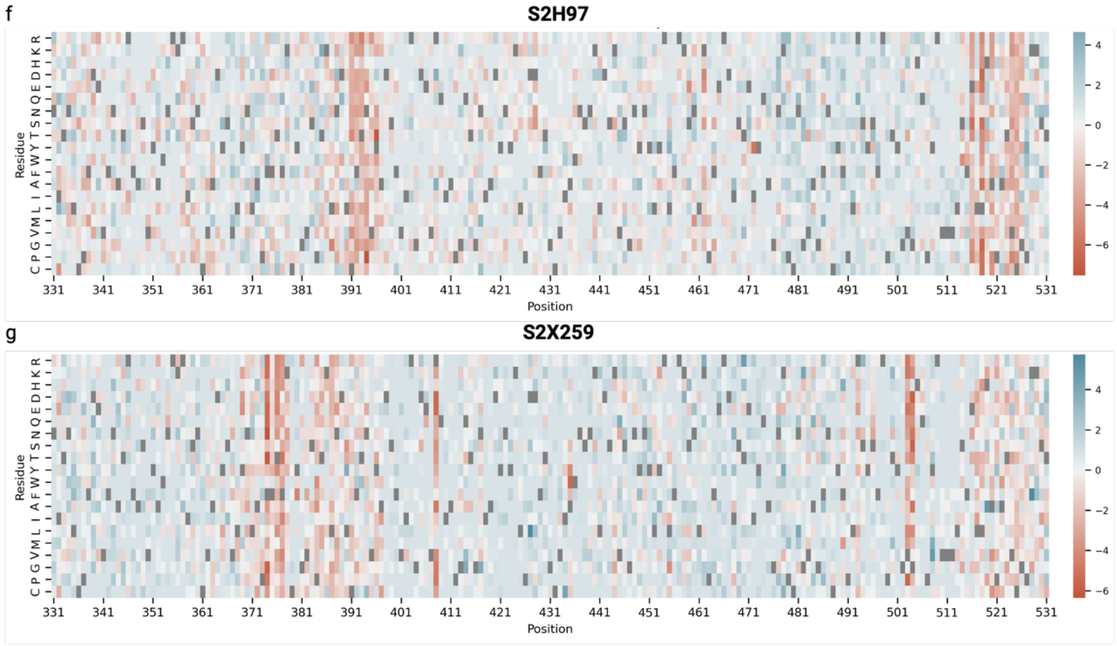

Supplementary Fig. 4 l Heatmaps showing binding scores per position across the RBD for libraries sorted against each antibody. Blue regions indicate mutations seen in greater frequency in the binding variant pool, while red regions indicate mutations with greater frequency in escape variants. WT (BA.1) residues are depicted by grey boxes.

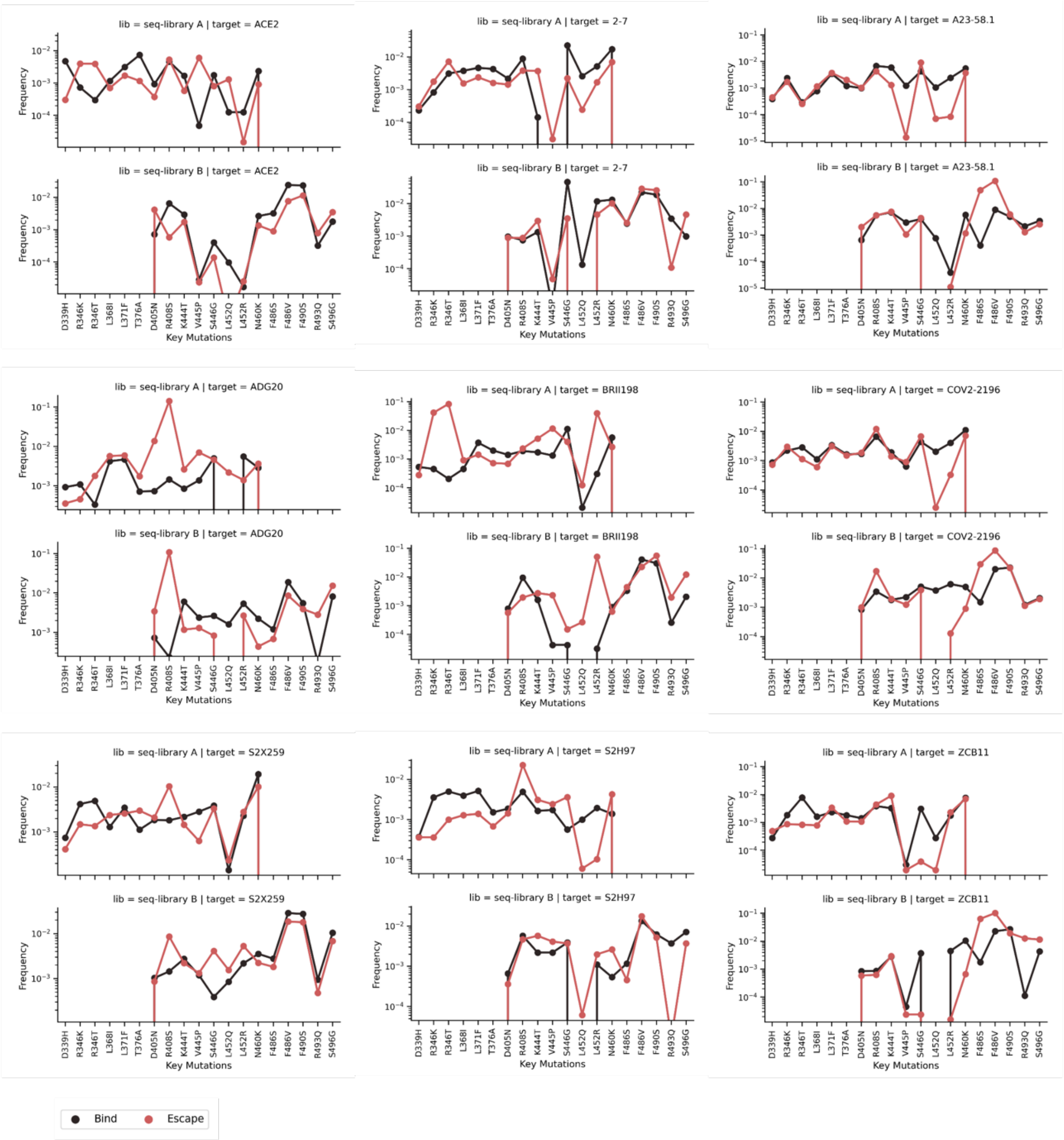

Supplementary Fig. 5 l Line plots show the frequencies of selected mutations in the binding and escape fractions of the deep sequencing data. The selected mutations have been observed in previously identified Omicron sublineages.

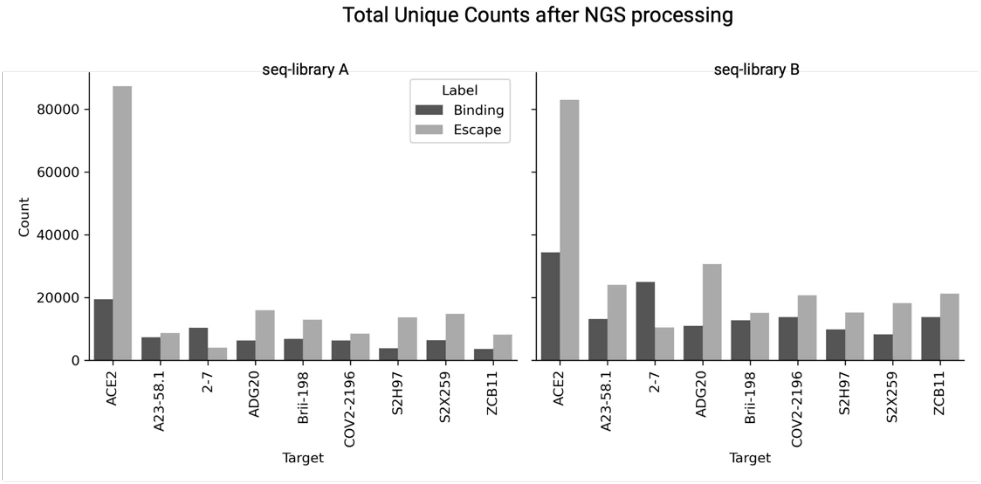

Supplementary Fig. 6 l Total unique sequences (aa) in each deep sequencing dataset (following pre-processing).

a

Unique sequences per distance seq-library A

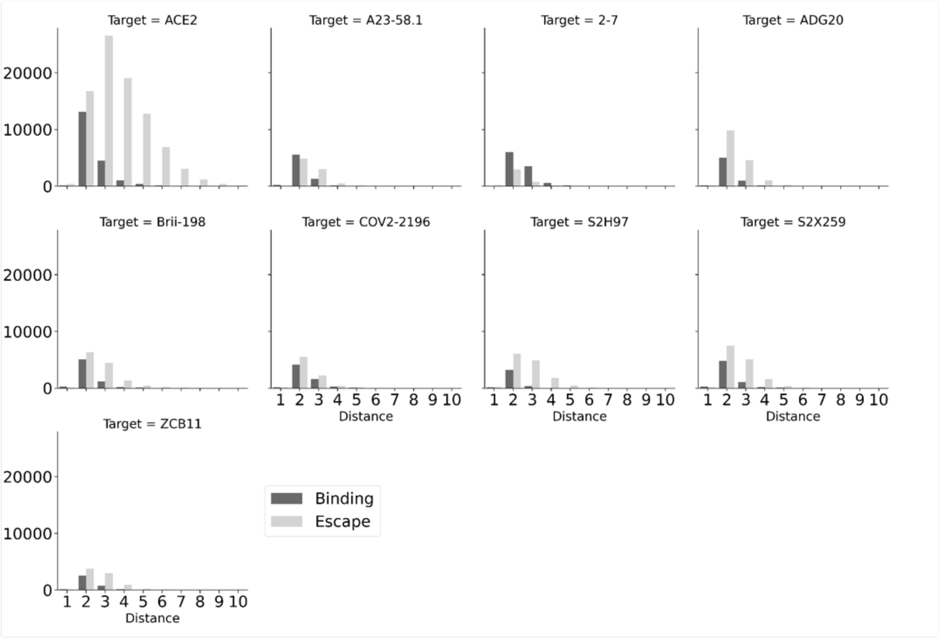

b

Unique sequences per distance seq-library B

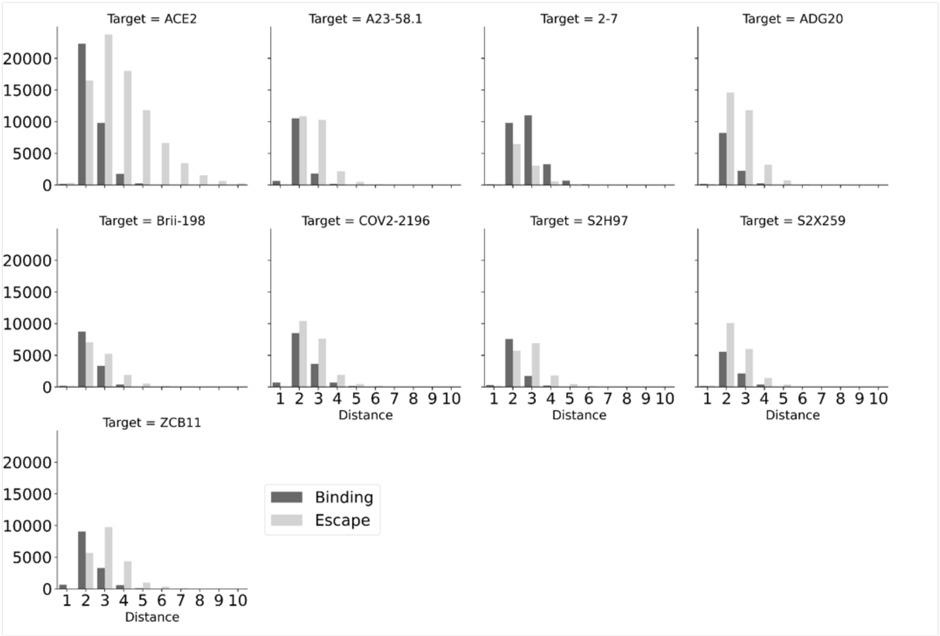

Supplementary Fig. 7 | Number of unique sequences (aa) in each dataset per ED from WT BA.1 RBD sequence. To allow visual comparison between datasets, the maximum of the y-axis in all antibody datasets has been set to the highest count in all datasets (20,000).

a

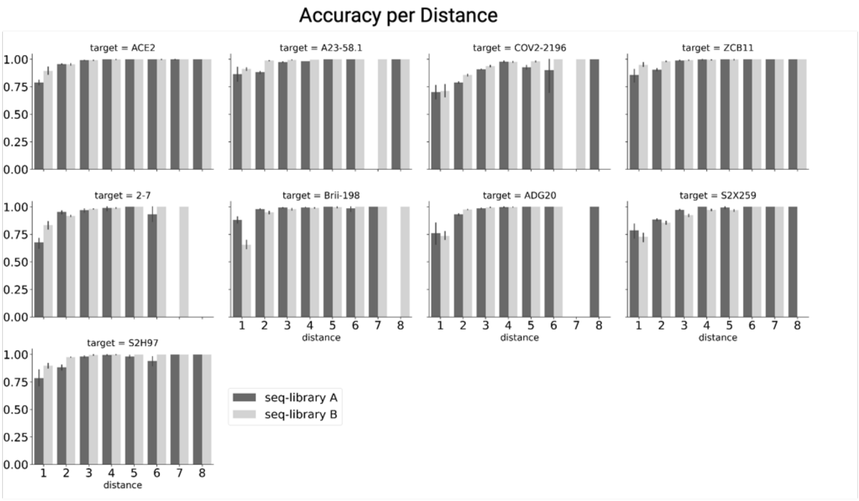

b

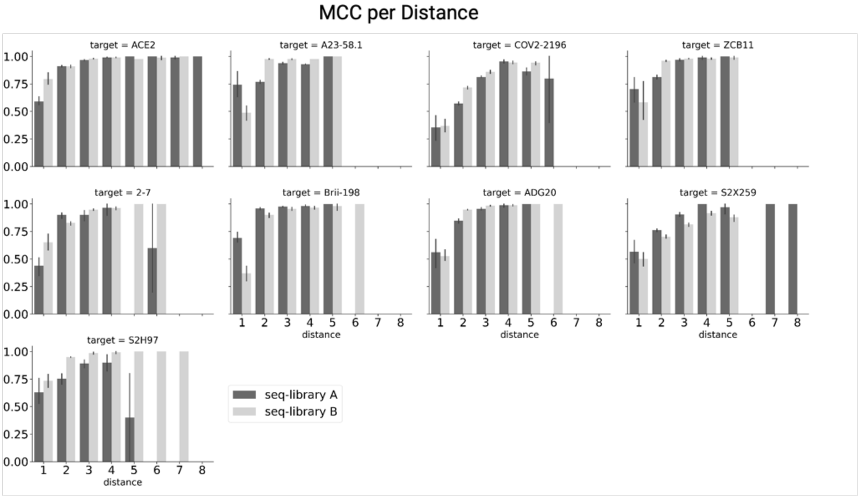

c

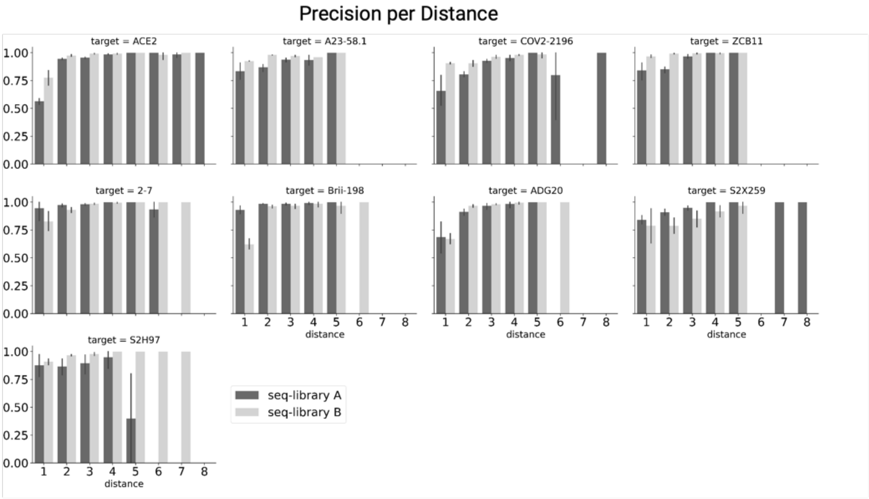

Supplementary Fig. 8 l CNN model performances on test sequences based on ED from BA.1; shown are**a**, accuracy, **b**, MCC, and **c**, precision. All scores shown are combined results from 5-fold cross-validation with a 80/10/10 train-val-test split, with error bars indicating SD.

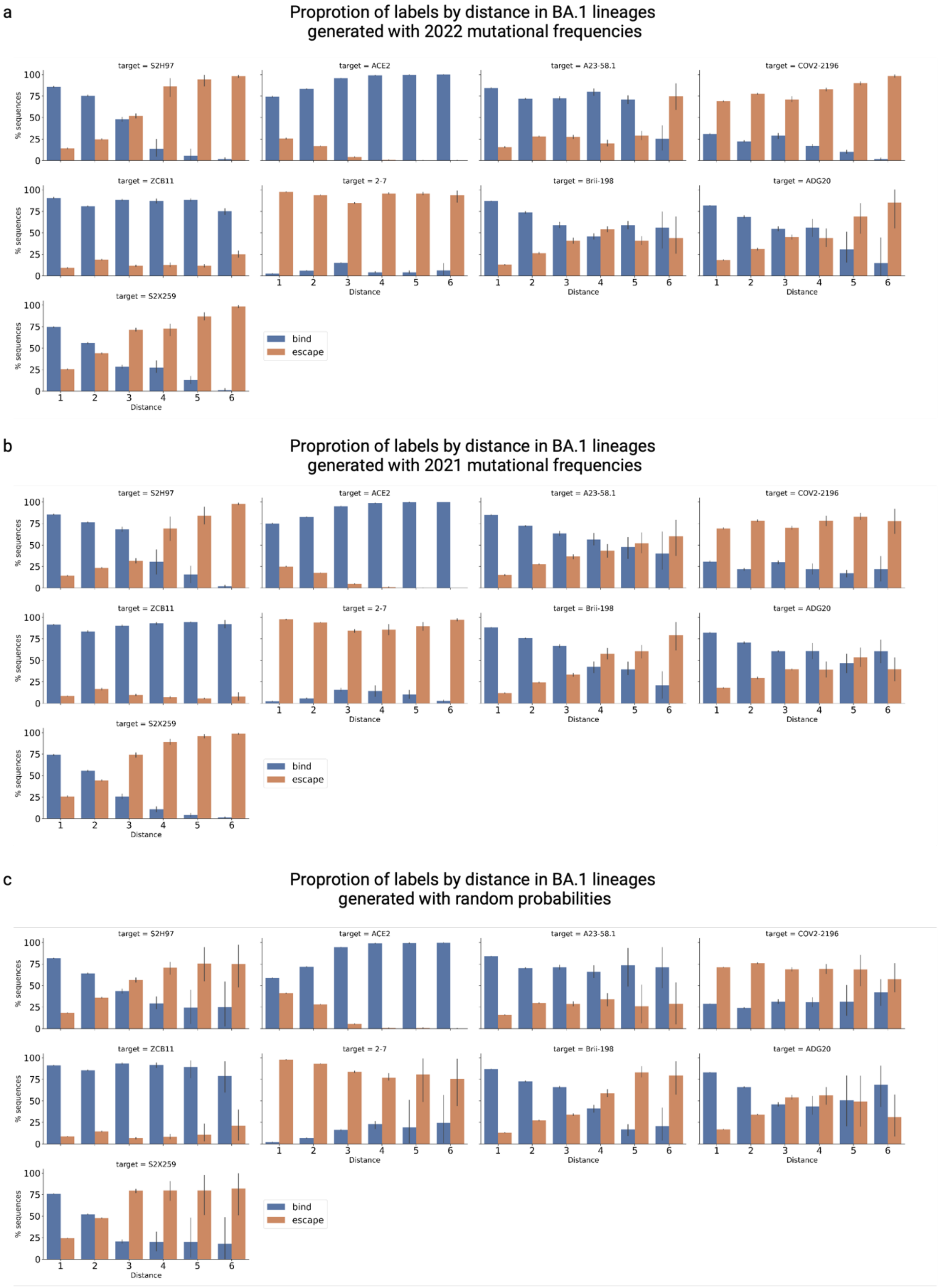

Supplementary Fig. 9 l Percent of predicted binding and escape variants per ED (from BA.1) for each antibody. Predictions were run on 10 sets of synthetic lineages: BA.1-derived lineages based on GISAID mutational frequencies from **a**, 2021, **b**, 2022 or **c**, randomized probabilities (see Methods). Each synthetic lineage contains up to 250,000 sequences. Error bars indicate SD.

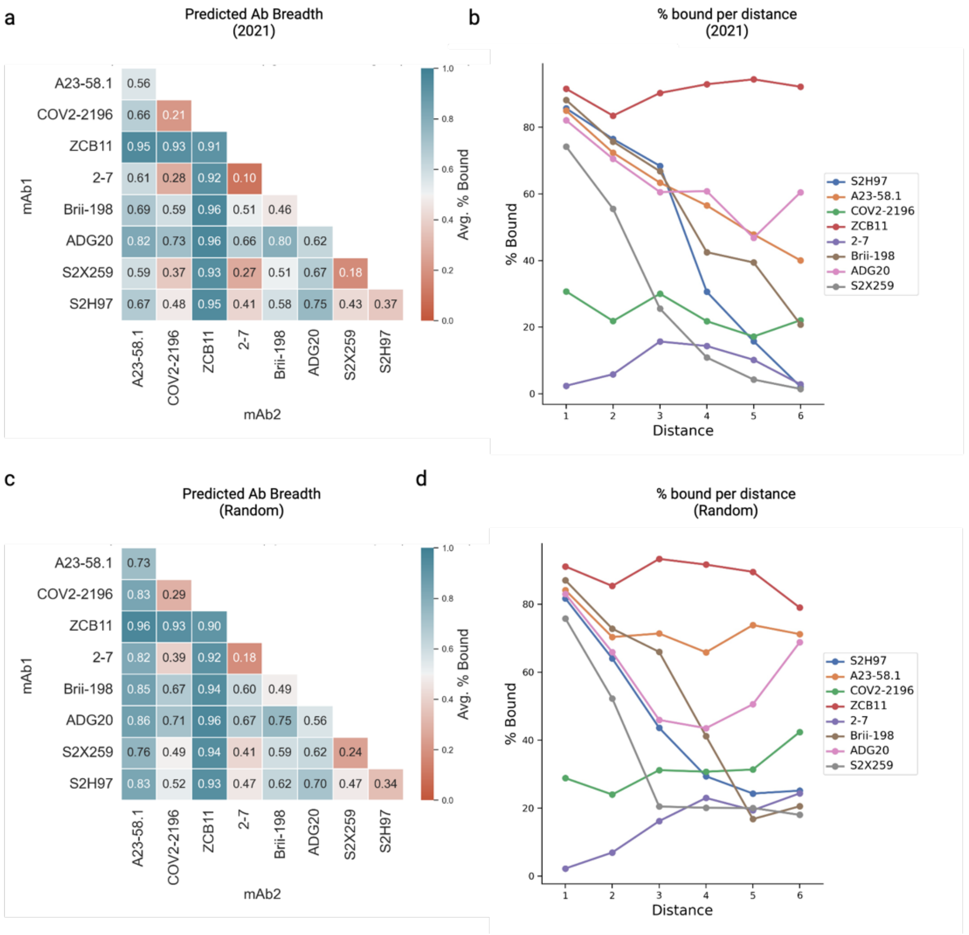

Supplementary Fig. 10 l a, predicted total antibody breadth and **b**, antibody breadth per ED (from BA.1) on synthetic lineages (BA.1-derived lineages based on 2021 GISAID mutational frequencies, see Methods). **c**, predicted total antibody breadth and **d**, antibody breadth per ED (from BA.1) on randomized synthetic lineages (BA.1-derived lineages based on uniform random sampling frequencies, see Methods).

Supplementary tables

	Sub-library 1	Sub-library 2	Sub-library 3	Sub-library 4
Fragment 1	tgatgatagctatcggcacacgtctcg**ctcc**AATATCACGAACCTTTGTCCTTTCGATGAGGTCTTCAATGCTACTAGAT**tcgc**agagacggaactgagtcggcgccgatg	tgatgatagctatcggcacacgtctcg**ctcc**AATATCACGAACCTTTGTCCTTTCGATGAGGTCTTCAATGCTACTAGATTCGCATCCGTG**tatg**cgagacggaactgagtcggcgccgatg	tgatgatagctatcggcacacgtctcg**ctcc**AATATCACGAACCTTTGTCCTTTCGATGAGGTCTTCAATGCTACTAGATTCGCATCCGTGTATGCATGGAATA**gaaa**ggagacggaactgagtcggcgccgatg	tgatgatagctatcggcacacgtctcg**ctcc**AATATCACGAACCTTTGTCCTTTCGATGAGG**tctt**cgagacggaactgagtcggcgccgatg
Fragment 2	gatagcggactttcggtcaacgtctct**tcgc**ATCCGTGTATGCATGGAATAGAAAGAGAATTAGTAATTGTGTAGCGGACT**acag**tgagacgtctgaatgtacaagcaaccc	gatagcggactttcggtcaacgtctcg**tatg**CATGGAATAGAAAGAGAATTAGTAATTGTGTAGCGGACTACTCTGTACTTTA**taac**tgagacgtctgaatgtacaagcaaccc	gatagcggactttcggtcaacgtctca**gaaa**GAGAATTAGTAATTGTGTAGCGGACTACAGTGTACTTTATAACTTGGCCC**cctt**cgagacgtctgaatgtacaagcaaccc	gatagcggactttcggtcaacgtctcg**tctt**CAATGCTACTAGATTCGCATCCGTGTATGCATGGAATAGAAAGAGAATTA**gtaa**tgagacgtctgaatgtacaagcaaccc
Fragment 3	aagtgggccgagcctggactcgtctct**acag**TGTACTTTATAACTTGGCCCCCTTCTTTACATTCAAGTGTTACGGTGTATC**tccc**agagacgcagctggttcctgcgtgagc	aagtgggccgagcctggactcgtctca**taac**TTGGCCCCCTTCTTTACATTCAAGTGTTACGGTGTATCTCCCACCAAGT**tgaa**tgagacgcagctggttcctgcgtgagc	aagtgggccgagcctggactcgtctcc**cctt**CTTTACATTCAAGTGTTACGGTGTATCTCCCACCAAGTTGAATGATCTAT**gctt**tgagacgcagctggttcctgcgtgagc	aagtgggccgagcctggactcgtctca**gtaa**TTGTGTAGCGGACTACAGTGTACTTTATAACTTGGCCCCCTTCTTTACAT**tcaa**ggagacgcagctggttcctgcgtgagc
Fragment 4	tcgagactcgggatgacagccgtctcc**tccc**ACCAAGTTGAATGATCTATGCTTTACAAACGTTTACGCCGATAGTTTCG**taat**tgagacgtcatagtacctcgggtacca	tcgagactcgggatgacagccgtctct**tgaa**TGATCTATGCTTTACAAACGTTTACGCCGATAGTTTCGTAATTAGAGGCG**atga**agagacgtcatagtacctcgggtacca	tcgagactcgggatgacagccgtctct**gctt**TACAAACGTTTACGCCGATAGTTTCGTAATTAGAGGCGATGAAGTGCGTC**agat**cgagacgtcatagtacctcgggtacca	tcgagactcgggatgacagccgtctct**tcaa**GTGTTACGGTGTATCTCCCACCAAGTTGAATGATCTATGCTTTACAAACG**ttta**cgagacgtcatagtacctcgggtacca
Fragment 5	acttactcaggttattgcttcgtctcg**taat**TAGAGGCGATGAAGTGCGTCAGATCGCACCAGGCCAGACGGGCAATATAGC**agat**tgagacggaacgcccatctagcggctg	acttactcaggttattgcttcgtctcg**atga**AGTGCGTCAGATCGCACCAGGCCAGACGGGCAATATAGCAGATTATAATT**ataa**ggagacggaacgcccatctagcggctg	acttactcaggttattgcttcgtctcc**agat**CGCACCAGGCCAGACGGGCAATATAGCAGATTATAATTATAAGCTGCCTGA**tgac**tgagacggaacgcccatctagcggctg	acttactcaggttattgcttcgtctcg**ttta**CGCCGATAGTTTCGTAATTAGAGGCGATGAAGTGCGTCAGATCGCACCAG**gcca**ggagacggaacgcccatctagcggctg
Fragment 6	gcgtcttgaatgctcggtcccgtctcc**agat**TATAATTATAAGCTGCCTGATGACTTCACCGGCTGTGTGATAGCTTGGAA**cagc**agagacggcttgcgaagtctacattgg	gcgtcttgaatgctcggtcccgtctct**ataa**GCTGCCTGATGACTTCACCGGCTGTGTGATAGCTTGGAACAGCAATAAAC**taga**tgagacggcttgcgaagtctacattgg	gcgtcttgaatgctcggtcccgtctca**tgac**TTCACCGGCTGTGTGATAGCTTGGAACAGCAATAAACTAGATTCCAAG**gtgt**cgagacggcttgcgaagtctacattgg	gcgtcttgaatgctcggtcccgtctcg**gcca**GACGGGCAATATAGCAGATTATAATTATAAGCTGCCTGATGACTTCACCGG**ctgt**ggagacggcttgcgaagtctacattgg

Fragment 7	tatatgaatgcgacctagaacgtctca**cagc**AATAAACTAGATTCCAAGGTGTCTGGCAATTACAATTATTTGTACCGTC**tgtt**cgagacgacggccgggaaaggtacgcg	tatatgaatgcgacctagaacgtctcc**taga**TTCCAAGGTGTCTGGCAATTACAATTATTTGTACCGTCTGTTCCGTAAAA**gcaa**tgagacgacggccgggaaaggtacgcg	tatatgaatgcgacctagaacgtctcg**gtgt**CTGGCAATTACAATTATTTGTACCGTCTGTTCCGTAAAAGCAATTTGAAAC**catt**tgagacgacggccgggaaaggtacgcg	tatatgaatgcgacctagaacgtctcg**ctgt**GTGATAGCTTGGAACAGCAATAAACTAGATTCCAAGGTGTCTGGCAAT**taca**agagacgacggccgggaaaggtacgcg
Fragment 8	cgcggtatgggaggatcaagcgtctcc**tgtt**CCGTAAAAGCAATTTGAAACCATTTGAAAGAGACATAAGCACTGAAATTT**acca**agagacggggccaatagagaggctcct	cgcggtatgggaggatcaagcgtctca**gcaa**TTTGAAACCATTTGAAAGAGACATAAGCACTGAAATTTACCAAGCAGGG**aaca**agagacggggccaatagagaggctcct	cgcggtatgggaggatcaagcgtctcc**catt**TGAAAGAGACATAAGCACTGAAATTTACCAAGCAGGGAACAAACCGTGCA**acgg**cgagacggggccaatagagaggctcct	cgcggtatgggaggatcaagcgtctct**taca**ATTATTTGTACCGTCTGTTCCGTAAAAGCAATTTGAAACCATTTGAAAGAGA**cata**agagacggggccaatagagaggctcct
Fragment 9	ctctcactcgctaggaggcacgtctct**acca**AGCAGGGAACAAACCGTGCAACGGCGTAGCTGGCTTTAACTGTTATTTCC**catt**agagacgaatgtaaaacaatggttact	ctctcactcgctaggaggcacgtctcg**aaca**AACCGTGCAACGGCGTAGCTGGCTTTAACTGTTATTTCCCATTAAGATCTTA**tag**ttgagacgaatgtaaaacaatggttact	ctctcactcgctaggaggcacgtctca**acgg**CGTAGCTGGCTTTAACTGTTATTTCCCATTAAGATCTTATAGTTTCAGACC**tacg**tgagacgaatgtaaaacaatggttact	ctctcactcgctaggaggcacgtctca**cata**AGCACTGAAATTTACCAAGCAGGGAACAAACCGTGCAACGGCGTAGCT**ggct**tgagacgaatgtaaaacaatggttact
Fragment 10	gcatcgatacataaaacatgcgtctcc**catt**AAGATCTTATAGTTTCAGACCTACGTATGGAGTCGGGCATCAGCCGTACC**gtgt**tgagacgctgtccatcggttgcccaaa	gcatcgatacataaaacatgcgtctca**tagt**TTCAGACCTACGTATGGAGTCGGGCATCAGCCGTACCGTGTTGTGGTTC**tttc**agagacgctgtccatcggttgcccaaa	gcatcgatacataaaacatgcgtctcc**tacg**TATGGAGTCGGGCATCAGCCGTACCGTGTTGTGGTTCTTTCATTTGAAC**tgct**ggagacgctgtccatcggttgcccaaa	gcatcgatacataaaacatgcgtctct**ggct**TTAACTGTTATTTCCCATTAAGATCTTATAGTTTCAGACCTACGTATGGA**gtcg**ggagacgctgtccatcggttgcccaaa
Fragment 11	gtgttaagtgtctatcaccccgtctcc**gtgt**TGTGGTTCTTTCATTTGAACTGCTGCACGCGCCCGCAACCGTATGCGGGCCGAAGAAATCAACG**gatt**agagacggtcgctgtactaatagttgt	gtgttaagtgtctatcaccccgtctcc**tttc**ATTTGAACTGCTGCACGCGCCCGCAACCGTATGCGGGCCGAAGAAATCAACG**gatt**agagacggtcgctgtactaatagttgt	gtgttaagtgtctatcaccccgtctcc**tgct**GCACGCGCCCGCAACCGTATGCGGGCCGAAGAAATCAACG**gatt**agagacggtcgctgtactaatagttgt	gtgttaagtgtctatcaccccgtctca**gtcg**GGCATCAGCCGTACCGTGTTGTGGTTCTTTCATTTGAACTGCTGCACGCGC**ccgc**agagacggggccgtttcccgcatataa
Fragment 12	-	-	-	acgccaggttgtatccgcatcgtctcc**ccgc**AACCGTATGCGGGCCGAAGAAATCAACG**gatt**agagacggtcgctgtactaatagttgt

Supplementary Table 1 | Sequences for fragments by sub-library. Sequences marked with uppercase letters are derived from the RBD open reading frame. The NNK codons are exclusively in this region. Bold lowercase sequences are the four nt homologies for GGA. The remaining lowercase sequences contain BsmBI recognition sites and primer binding sites for double strand synthesis.

Therapeutic antibodies	Concentration [nM]
S2X259	12.5
S2H97	2.5
COV2-2196	12.5
ZCB11	6.25
2-7	60
ADG20	7.5
A23-58.1	5
Brii-198	10

Supplementary Table 2 I Antibody concentrations used for FACS of yeast displayed RBD libraries. The concentrations were determined based on the titration curves shown in **Supplementary Fig. 3**.

Population	Antigen	Paired and filtered Reads: Binding	Paired and filtered Reads: Non-Binding
seq-Library A	ACE2	1.92E+06	1.75E+06
seq-Library B	ACE2	2.32E+06	1.60E+06
seq-Library A, ACE2 binding	2-7	3.27E+05	5.52E+05
seq-Library A, ACE2 binding	A23-58.1	1.20E+06	1.08E+06
seq-Library A, ACE2 binding	ADG20	8.37E+05	8.83E+05
seq-Library A, ACE2 binding	Brii-198	9.40E+05	3.59E+05
seq-Library A, ACE2 binding	COV2-2196	5.59E+05	9.40E+05
seq-Library A, ACE2 binding	S2H97	5.37E+05	7.23E+05
seq-Library A, ACE2 binding	S2X259	6.22E+05	5.59E+05
seq-Library A, ACE2 binding	ZCB11	5.52E+05	8.30E+05
seq-Library B, ACE2 binding	2-7	1.02E+06	9.32E+05
seq-Library B, ACE2 binding	A23-58.1	9.12E+05	7.38E+05
seq-Library B, ACE2 binding	ADG20	9.38E+05	9.03E+05
seq-Library B, ACE2 binding	Brii-198	9.70E+05	5.48E+05
seq-Library B, ACE2 binding	COV2-2196	7.66E+05	9.70E+05
seq-Library B, ACE2 binding	S2H97	8.42E+05	4.43E+05
seq-Library B, ACE2 binding	S2X259	7.50E+05	7.66E+05
seq-Library B, ACE2 binding	ZCB11	9.32E+05	6.09E+05

Supplementary Table 3 l Deep sequencing statistics for sorted RBD libraries

Parameter	MLP	ProtCNN	ProtCNN-Final
Learning Rate	0.01 - 0.0001		**0.0001**
Optimizer	Adam, SGD		**Adam**
Minority Ratio (dataset balance)	0.1 - 0.5		**0.25**
Epochs	15 - 75		**40**
Test/Val ratio	0.1-0.25		**0.2**
MLP Parameters			
Dense Dimensions	32 - 512		
# Dense Layers	1-3		
Dense Dropout	0 - 0.5		
CNN Parameters			
Kernel Size		3 - 21	**15**
Stride		1-3	**2**
Filter Number		32 - 512	**128**
Padding		"Same", 1	**"Same"**
Pool Size		1-3	**2**
Pool Stride		1-3	**1**
Dilation Rate		2-5	**3**
Residual Blocks		1-3	**2**

Supplementary Table 4 l Hyperparameter search conditions for CNN and MLP models, and final parameters used for CNN models.

Seq-library A									
Score	ACE2	A23-58.1	COV2-2196	ZCB11	2-7	Brii-198	ADG20	S2X259	S2H97
Accuracy	0.983±0.001	0.923±0.001	0.83±0.001	0.939±0.009	0.964±0.007	0.985±0.003	0.955±0.002	0.926±0.002	0.936±0.012
F1	0.956±0.002	0.92±0.001	0.785±0.004	0.91±0.011	0.975±0.004	0.978±0.005	0.925±0.003	0.881±0.001	0.869±0.018
MCC	0.946±0.002	0.848±0.001	0.653±0	0.869±0.015	0.911±0.017	0.967±0.008	0.894±0.003	0.829±0.001	0.835±0.021
Precision	0.939±0.001	0.892±0.015	0.862±0.012	0.859±0.032	0.971±0.016	0.977±0.002	0.891±0.02	0.888±0.031	0.795±0.043
Recall	0.974±0.003	0.949±0.018	0.722±0.016	0.969±0.019	0.98±0.008	0.979±0.01	0.961±0.017	0.876±0.033	0.962±0.02

Seq-library B									
Model	ACE2	A23-58.1	COV2-2196	ZCB11	2-7	Brii-198	ADG20	S2X259	S2H97
Accuracy	0.985±0.005	0.992±0.001	0.9±0.003	0.989±0.001	0.952±0.002	0.963±0.003	0.983±0	0.872±0.02	0.983±0.001
F1	0.974±0.008	0.989±0.002	0.871±0.004	0.986±0.001	0.966±0.001	0.96±0.003	0.97±0	0.82±0.018	0.979±0.001
MCC	0.962±0.004	0.983±0.002	0.791±0.006	0.977±0.002	0.884±0.004	0.926±0.005	0.959±0.001	0.74±0.022	0.965±0.002
Precision	0.98±0.006	0.985±0.003	0.902±0.006	0.981±0.003	0.956±0.002	0.941±0.01	0.958±0.002	0.732±0.052	0.966±0.006
Recall	0.969±0.021	0.994±0.001	0.842±0.002	0.991±0.002	0.976±0.002	0.98±0.006	0.983±0.002	0.938±0.039	0.993±0.003

Supplementary Table 5 I Metrics of final CNN models trained on ACE2 binding and antibody escape. All metrics were evaluated using 5x cross-validation splits.

Primer name	Sequence (5’ to 3’)
seq-library A fwd	TCGTCGGCAGCGTCAGATGTGTATAAGAGACAGGATGTGCCCGATTATGCG
seq-library A rev	GTCTCGTGGGCTCGGAGATGTGTATAAGAGACAGGCCGTTGCACGGTTTGTT
seq-library B fwd	TCGTCGGCAGCGTCAGATGTGTATAAGAGACAGGTGTTACGGTGTATCTCCC
seq-library B rev	GTCTCGTGGGCTCGGAGATGTGTATAAGAGACAGCTCACTTGTCATCATCGTCC

Supplementary Table 6 I Primers used to amplify seq-libraries A and B in a targeted fashion for subsequent deep sequencing.
