# Supplementary material for: Deep mutational learning for the selection of therapeutic antibodies resistant to the evolution of Omicron variants of SARS-CoV-2
Source: Nat Biomed Eng. 2025 Mar 5;9(4):552–65. doi: 10.1038/s41551-025-01353-4 (PMC12003156; doi:10.1038/s41551-025-01353-4)
Supplement: Supplementary file 2 — Reporting Summary [file 41551_2025_1353_MOESM2_ESM.pdf]

nature portfolio

Corresponding author(s): Sai Reddy

Last updated by author(s): Sep 5, 2024

Reporting Summary

Nature Portfolio wishes to improve the reproducibility of the work that we publish. This form provides structure for consistency and transparency in reporting. For further information on Nature Portfolio policies, see our Editorial Policies and the Editorial Policy Checklist.

Statistics

For all statistical analyses, confirm that the following items are present in the figure legend, table legend, main text, or Methods section.

n/a	Confirmed	
☐	☒	The exact sample size (*n*) for each experimental group/condition, given as a discrete number and unit of measurement
☐	☒	A statement on whether measurements were taken from distinct samples or whether the same sample was measured repeatedly
☒	☐	The statistical test(s) used AND whether they are one- or two-sided<br>*Only common tests should be described solely by name; describe more complex techniques in the Methods section.*
☒	☐	A description of all covariates tested
☒	☐	A description of any assumptions or corrections, such as tests of normality and adjustment for multiple comparisons
☒	☐	A full description of the statistical parameters including central tendency (e.g. means) or other basic estimates (e.g. regression coefficient) AND variation (e.g. standard deviation) or associated estimates of uncertainty (e.g. confidence intervals)
☒	☐	For null hypothesis testing, the test statistic (e.g. *F*, *t*, *r*) with confidence intervals, effect sizes, degrees of freedom and *P* value noted<br>*Give P values as exact values whenever suitable.*
☒	☐	For Bayesian analysis, information on the choice of priors and Markov chain Monte Carlo settings
☒	☐	For hierarchical and complex designs, identification of the appropriate level for tests and full reporting of outcomes
☒	☐	Estimates of effect sizes (e.g. Cohen's *d*, Pearson's *r*), indicating how they were calculated

Our web collection on statistics for biologists contains articles on many of the points above.

Software and code

Policy information about availability of computer code

Data collection	Illumina software and FASTQC were used for the initial processing and quality assessment of NGS datasets.
Data analysis	Custom code written in Python and R have been used for data analysis. The code associated with the work in this study is available at https://github.com/LSSI-ETH/Omicron_DML.

For manuscripts utilizing custom algorithms or software that are central to the research but not yet described in published literature, software must be made available to editors and reviewers. We strongly encourage code deposition in a community repository (e.g. GitHub). See the Nature Portfolio guidelines for submitting code & software for further information.

Data

Policy information about availability of data

All manuscripts must include a data availability statement. This statement should provide the following information, where applicable:

- Accession codes, unique identifiers, or web links for publicly available datasets
- A description of any restrictions on data availability
- For clinical datasets or third party data, please ensure that the statement adheres to our policy

The main data supporting the results in this study are available within the paper and its Supplementary Information. Source data are provided with this paper. The processed datasets generated during the study are available at https://zenodo.org/records/11172179.

Research involving human participants, their data, or biological material

Policy information about studies with human participants or human data. See also policy information about sex, gender (identity/presentation), and sexual orientation and race, ethnicity and racism.

Reporting on sex and gender	The study did not involve human research participants.
Reporting on race, ethnicity, or other socially relevant groupings	–
Population characteristics	–
Recruitment	–
Ethics oversight	–

Note that full information on the approval of the study protocol must also be provided in the manuscript.

Field-specific reporting

Please select the one below that is the best fit for your research. If you are not sure, read the appropriate sections before making your selection.

☒ Life sciences ☐ Behavioural & social sciences ☐ Ecological, evolutionary & environmental sciences

For a reference copy of the document with all sections, see nature.com/documents/nr-reporting-summary-flat.pdf

Life sciences study design

All studies must disclose on these points even when the disclosure is negative.

Sample size	8 different monoclonal antibodies and human ACE2 were investigated for binding/non-binding to a library of Omicron BA.1 variants.<br>E. coli and yeast library sizes were determined by dilution plating.
Data exclusions	No data were excluded.
Replication	The methods and materials used for data generation, collection and analysis are described in detail in Methods and were reproducible.
Randomization	Sequences selected during the training and assessment of models were randomly subsampled from the full experimental datasets. No other randomization was relevant or required for the remaining experiments performed.
Blinding	Blinding was not relevant.

Reporting for specific materials, systems and methods

We require information from authors about some types of materials, experimental systems and methods used in many studies. Here, indicate whether each material, system or method listed is relevant to your study. If you are not sure if a list item applies to your research, read the appropriate section before selecting a response.

Materials & experimental systems

n/a	Involved in the study	
☐	☒	Antibodies
☐	☒	Eukaryotic cell lines
☒	☐	Palaeontology and archaeology
☒	☐	Animals and other organisms
☒	☐	Clinical data
☒	☐	Dual use research of concern
☒	☐	Plants

Methods

n/a	Involved in the study	
☒	☐	ChIP-seq
☐	☒	Flow cytometry
☒	☐	MRI-based neuroimaging

Antibodies

Antibodies used	A23-58.1, COV2-2196, Brii-198, ZCB11, 2-7, S2X259, ADG20, S2H97 anti-human IgG-AF647 (Jackson Immunoresearch 109-605-098), anti-FLAG Tag APC (Biolegend 637308).

Validation	Antibodies produced in-house were validated by testing for binding to the Omicron BA.1 sequence.<br>Antibodies purchased from BioLegend were quality-control tested by intracellular immunofluorescent staining with flow cytometric analysis.

Eukaryotic cell lines

Policy information about cell lines and Sex and Gender in Research

Cell line source(s)	EBY100 (ATCC).
Authentication	The EBY100 yeast cell line was authenticated via the successful cell-surface display of RBD.
Mycoplasma contamination	The cells tested negative for mycoplasma contamination.
Commonly misidentified lines<br>(See ICLAC register)	No commonly misidentified cell lines were used.

Flow Cytometry

Plots

Confirm that:

☒ The axis labels state the marker and fluorochrome used (e.g. CD4-FITC).

☒ The axis scales are clearly visible. Include numbers along axes only for bottom left plot of group (a 'group' is an analysis of identical markers).

☒ All plots are contour plots with outliers or pseudocolor plots.

☒ A numerical value for number of cells or percentage (with statistics) is provided.

Methodology

Sample preparation	The Omicron RBD library was displayed using the yeast strain EBY100. Cells were grown for 1 day in selective medium at 30 °C before inducing surface display for 2 days at 23 °C. After completion of induction, the cells were washed before incubation with ACE2/monoclonal antibody for 1 h at 4 °C. The cells were washed before incubation with anti-FLAG-PE and anti-IgG-AF647. The cells were washed once more before sorting.
Instrument	Flow cytometry was performed using BD FACSAria Fusion (5 lasers configurations, UV, 488, 405, 445, 561, 640) and BD Influx (8 lasers configurations, 488nm, 405nm, 445nm, 514nm, 532nm, 561nm, 594nm and 640nm).
Software	The data were analysed using FlowJo v10.9.
Cell population abundance	The post-sort purity of populations was determined by flow-cytometry analysis following expansion of the collected populations. 2 or 3 rounds of sorting were necessary to isolate pure populations.
Gating strategy	The FSC/SSC gate showed a single population. An FSC-H/FSC-W gate was used to distinguish singlets from doublets. Cell doublets were excluded. RBD expressing cells were then identified by their fluorescence signal from the 561 nm laser (586/15 nm band-pass filter, 570 nm longpass filter). Cells expressing antibody binding RBD variants were then identified and gated determined by their fluorescence signal from 633 nm laser (670/14 nm band-pass filter). Boundaries between positive and negative populations were determined by including the following control samples in the analysis: 1) a yeast line expressing wild-type Omicron BA.1 RBD stained with either ACE2 or monoclonal antibody. 2) a yeast line expressing wild-type Omicron BA.1 RBD stained only for expression and not with ACE2/monoclonal antibody.<br><br>The population with intermediate binding signal and the double negative population were not collected.

☒ Tick this box to confirm that a figure exemplifying the gating strategy is provided in the Supplementary Information.
